# Targeting lymphatic function in cardiovascular-kidney-metabolic syndrome: preclinical methods to analyze lymphatic function and therapeutic opportunities

**DOI:** 10.3389/fcvm.2024.1412857

**Published:** 2024-06-10

**Authors:** Joseph Wayne M. Fowler, LouJin Song, Kelly Tam, Rachel J. Roth Flach

**Affiliations:** Internal Medicine Research Unit, Pfizer Research and Development, Cambridge, MA, United States

**Keywords:** lymphatics, drug discovery, pharmacology, preclinical model of cardiovascular disease, cardiovascular-kidney-metabolic syndrome (CKM)

## Abstract

The lymphatic vascular system spans nearly every organ in the body and serves as an important network that maintains fluid, metabolite, and immune cell homeostasis. Recently, there has been a growing interest in the role of lymphatic biology in chronic disorders outside the realm of lymphatic abnormalities, lymphedema, or oncology, such as cardiovascular-kidney-metabolic syndrome (CKM). We propose that enhancing lymphatic function pharmacologically may be a novel and effective way to improve quality of life in patients with CKM syndrome by engaging multiple pathologies at once throughout the body. Several promising therapeutic targets that enhance lymphatic function have already been reported and may have clinical benefit. However, much remains unclear of the discreet ways the lymphatic vasculature interacts with CKM pathogenesis, and translation of these therapeutic targets to clinical development is challenging. Thus, the field must improve characterization of lymphatic function in preclinical mouse models of CKM syndrome to better understand molecular mechanisms of disease and uncover effective therapies.

## 1 Lymphatic vascular biology

The vascular system plays a critical role in maintaining normal function of the human body, and it is made up of blood and lymphatic vasculature. As a complement to the blood vasculature that delivers oxygen and nutrients to tissues, the lymphatic vasculature consists of blind-ended vessels that form a unidirectional network to transport interstitial fluids, metabolites, and immune cells to secondary lymphoid organs and eventually back to the blood circulation (1). The blinded-ended vessels, or the lymphatic capillaries reside in the tissue, and they demonstrate discontinuous button-like junctions, which allow them to absorb tissue fluid and transport immune cells towards the collecting vessels (2). The lymphatic collecting vessels, on the other hand, have zipper-like junctions and contain valves, which allow a unidirectional transport of lymph against gravity towards the blood circulation (3). In addition to the distinct morphological features and function of lymphatic vessels, lymphatic endothelium is also distinguished from blood endothelium by the high expression of multiple lymphatic specific regulators, including prospero homeobox protein 1 (PROX1) and vascular endothelial growth factor receptor-3 (VEGFR3) (4). PROX1 is a key transcriptional regulator for lymphatic endothelial cell fate and identity, while VEGFR3 is the principal receptor for lymphangiogenesis (5). There are two well-known ligands for VEGFR3, which are vascular endothelial growth factor C (VEGFC) and vascular endothelial growth factor D (VEGFD), and the activation of VEGFR3 upon its binding to the ligands induces lymphangiogenesis (6). Moreover, lymphatic capillaries can also be distinguished from collecting vessels by the high expression of lymphatic vessel endothelial hyaluronan receptor type 1 (LYVE1) and chemokine (C-C motif) ligand 19 and 21 (CCL19/CCL21) (7). CCL19 and CCL21 are important in the interaction between lymphatic capillaries and C-C Chemokine Receptor 7 (CCR7)-expressing dendritic cells (DCs) (8). Although often overlooked compared to the blood vasculature, the lymphatic vasculature plays an important role in health and disease, and increasing evidence suggests that the lymphatics could be a potential new therapeutic target for cardiovascular-kidney-metabolic (CKM) syndrome.

## 2 CKM syndrome and lymphatics

**Figure 1:**
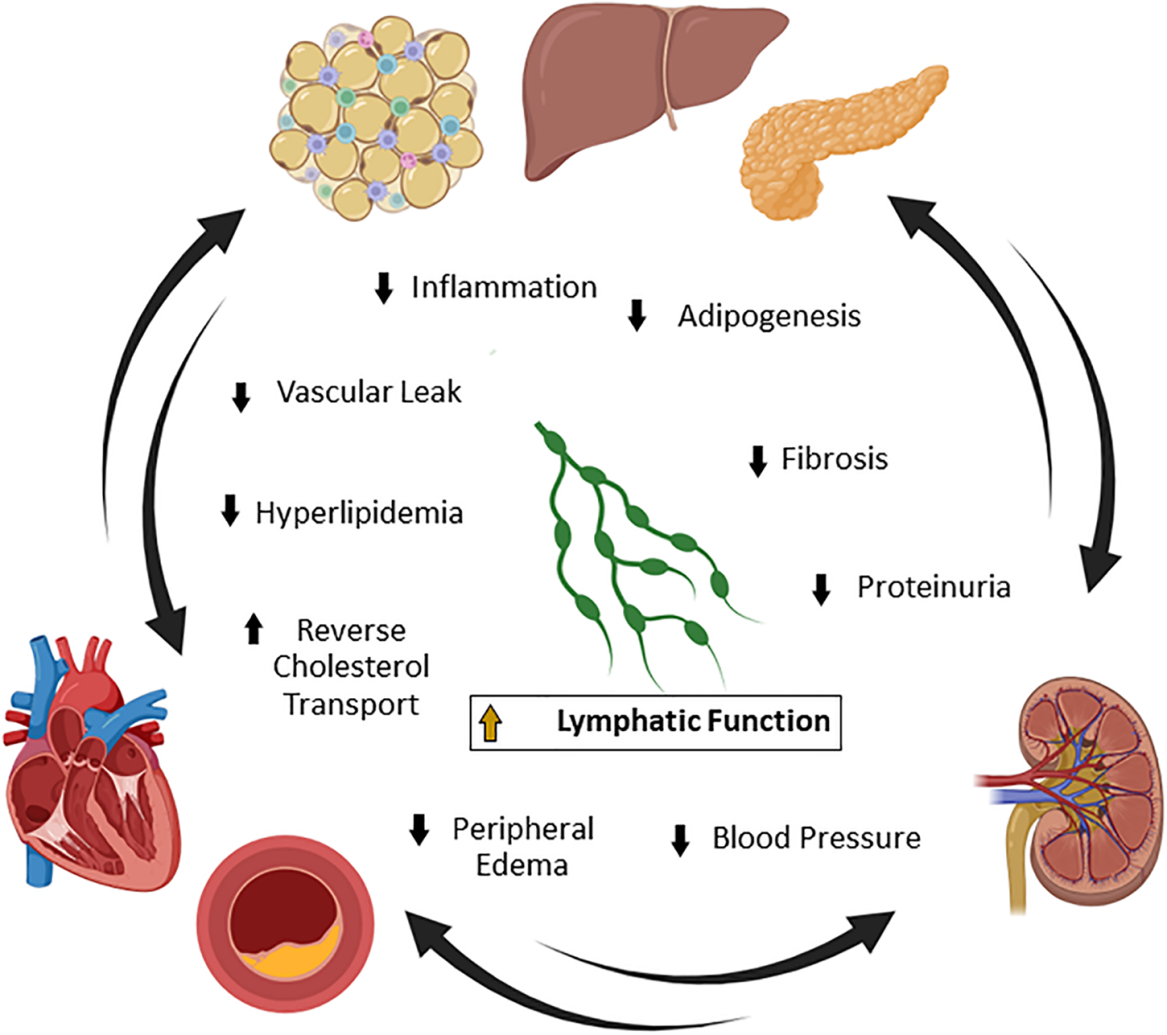
Lymphatic function and cardiovascular-kidney-metabolic (CKM) syndrome. CKM syndrome is a complex disease involving connected pathologies between metabolism, the cardiovascular network, and the renal system. Lymphatic vascular homeostasis is important for the maintenance of systemic lipid and metabolite levels, blood pressure, fluid transport, and immune trafficking. Many of these pathways contribute to the manifestation of CKM syndrome in mice and humans. Improvement of lymphatic function is a potentially novel strategy to simultaneously treat multiple drivers of pathology in CKM syndrome by reducing obesity, inflammation, fibrosis, proteinuria, blood pressure, and edema. Created with BioRender.com.

CKM syndrome is the clinical concept that there is a connected pathology between cardiovascular disease, kidney disease and metabolic diseases such as obesity and diabetes, and that new approaches are necessary to treat the diseases as a whole entity (9). CKM syndrome is characterized by early dysfunctional adiposity that can progress to hyperglycemia and insulin resistance (10). This predisposes patients to systemic inflammatory and oxidative stresses, which, when combined with genetic and environmental factors, cascades into cardiorenal dysfunction (11, 12). At this point, the cardiovascular system, kidney, and systemic metabolic network cascade through a series of communications that ultimately lead to coronary artery disease, peripheral artery disease, stroke, heart failure, or renal failure (9). Improving the lymphatic vascular network, which spans throughout multiple organs and regulates several aspects of disease, may provide a novel therapeutic avenue for the treatment of CKM syndrome (Figure 1). The lymphatic vasculature has been shown in numerous studies to be dysfunctional in heart failure, atherosclerosis, kidney disease, and obesity that selective improvement of lymphatics can provide preclinical efficacy in individual models within CKM syndrome (13–15). However, the application of improving lymphatic vascular function has had limited attempts clinically. We outline here various reports of lymphatic vascular involvement in CKM health, techniques to directly interrogate lymphatic function for drug discovery, therapeutic pathways that may have clinical benefit in CKM, and the challenges of translating these approaches to clinical development.

### 2.1 Heart failure

Heart failure is a complex syndrome with heterogenous clinical phenotypes, various underlying etiologies, and mechanisms of impairment (16, 17). Despite the heterogeneity, anti-hypertensive thiazide diuretics, along with angiotensin-converting enzyme (ACE) inhibitors and angiotensin receptor blockers, remain one of the most commonly used medications for heart failure, especially in patients with elevated risk factors (18). This provides clear rationale for the improvement of lymphatic function in this large patient population as the lymphatic network controls the transport of interstitial fluid throughout the body (19). Second, pulmonary edema, which involves the buildup of fluid in the lungs, is a common symptom of congestive heart failure and results from increased venous and capillary pressure to the lungs secondary to the increase in left ventricular pressure (20). It has been suggested that about 80% of patients with congestive heart failure have pulmonary edema, which worsens patient symptoms, including exacerbated shortness of breath, chest pain, and fatigue (21). Increasing lymphatic function may directly enhance patient quality of life by clearing both peripheral and pulmonary interstitial fluid and absolving these symptoms. Among the heart failure sub-categories, heart failure with preserved ejection fraction (HFpEF) has become the most common form and is characterized by preserved ejection fraction and diastolic dysfunction (22). Patients with HFpEF demonstrated peripheral lymphatic rarefaction and reduced lymphatic drainage, suggesting a potential association between lymphatics and HFpEF etiology (23, 24).

Many recent studies have established a clear role for the lymphatic vasculature in rodent models of heart failure. In preclinical models of myocardial infarction, robust lymphangiogenesis was observed in injured heart and potentially represents an endogenous response to alleviate cardiac injury (25, 26). Consistently, in the setting of chronic heart failure induced by pressure overload, lymphangiogenesis was also observed in heart, although to a lesser extent (27, 28). Additionally, cardiac lymphatic dysfunction in mice induced diastolic dysfunction, a hallmark of HFpEF, and cardiac lymphangiogenesis stimulated by cell therapy restored diastolic dysfunction, supporting a potential therapeutic role of lymphatics in HFpEF (29). More interestingly, human heart failure cardiac tissues showed evidence of increased lymphatic density (27). However, these lymphatics displayed reduced lumen diameter, suggesting that although the heart can endogenously compensate in this disease, it may be insufficient to attenuate cardiac dysfunction (27). Moreover, administration of lymphangiogenic factors such as VEGFC_C156S_, a point mutant that is a specific VEGFR3 ligand, to further facilitate lymphangiogenesis and improve lymphatic function in rodent model of heart failure demonstrated beneficial effects, as we discuss in greater detail in another section of this review article, highlighting the potential of targeting lymphatics for the treatment of heart failure (30, 31).

### 2.2 Atherosclerosis

Emerging evidence indicates an important role of the lymphatic vasculature in the progression and regression of atherosclerotic plaques in large arteries. Robust lymphangiogenesis has been reported in aortas and coronary arteries of atherosclerotic mice and humans and correlates with atherosclerosis severity (32, 33). As atherosclerosis progresses, the lymphatic network expands along the aorta, forming a loose vascular plexus and breaching only as far as the adventitial layer of the vessel (33). Although mainly studied in rodent models of atherosclerosis, the lymphatic vasculature is thought to play two important roles in atherosclerosis: (1) control of systemic lipoprotein levels and (2) lymphatic clearance of plaque lipids and immune cells (33–36). The lymphatic system is highly important in the regulation of dietary lipid uptake as well as peripheral lipoprotein trafficking and can control levels of pro-atherogenic low-density lipoprotein (LDL) and athero-protective high-density lipoprotein (HDL) (37, 38). Soluble Vegfr3-expressing or Vegfr3-mutant (Chy) mice were crossed with low-density lipoprotein receptor, *Ldlr*, knockout mice, which led to significantly increased total serum cholesterol and triglycerides due to an increase in circulating very low-density lipoprotein (VLDL) and LDL (36). Furthermore, Vegfr3-specific agonism via VEGFC_C156S_ significantly improved lymphatic function and lowered circulating LDL (35). Rodent atherosclerosis is highly dependent on circulating pro-atherogenic lipoprotein particles, and both studies showed that improved lymphatic function correlated with decreased LDL and decreased atherosclerotic burden (35, 36). Secondly, the lymphatic vasculature that forms on the outer edges of the growing atherosclerotic plaque may have a direct function of clearing lipids and immune cells as well as serving as an important conduit for reverse cholesterol transport. Martel et al. showed that the lymphatic vasculature directly transports cholesterol from the plaque to acceptor HDL in circulation, and inhibition of Vegfr3 signaling caused aortic cholesterol retention (34). Lastly, this aortic lymphatic vascular network is critical for the regression of atherosclerosis in mice given ezetimibe, which prevents cholesterol absorption (33). Enhancing lymphatic clearance and overall function may be a novel and effective strategy to reduce cardiovascular-related death beyond traditional lipid-lowering therapies.

### 2.3 Chronic kidney disease

Renal lymphatics, a largely neglected target in discussions regarding kidney diseases, has also drawn more attention in the past few years. There are abundant lymphatic vessels in kidney cortex of all species, which play a critical role in maintaining tissue homeostasis and regulating interstitial edema (13, 39). Similar to heart failure and atherosclerosis, robust lymphangiogenesis has been observed in preclinical models of acute kidney injury (AKI) and chronic kidney disease (CKD) as well as kidney biopsies from patients with AKI, nephropathy and diabetic kidney disease (DKD) (40–42). Increased lymphatic density was often observed at the site of tubulointerstitial lesions and areas of fibrosis and inflammation, suggesting that local lymphangiogenesis may represent an intrinsic response to resolve tissue damage, fibrosis and local inflammation (43, 44). In addition, reduced lymphatic vessel diameter and branching was observed in the kidney of an autosomal dominant polycystic kidney disease (ADPKD) mouse model, indicating that lymphatic function might be impaired in the pathogenesis of ADPKD (45). More interestingly, enhancing lymphangiogenesis in kidney through local Vegfd overexpression, using a genetic approach or kidney targeted nanoparticles, reduced blood pressure, increased sodium excretion and alleviated renal inflammation in hypertensive mouse models, implicating that renal lymphatics also contribute to hypertension and hypertensive kidney disease (46–48). Although there has been recent interrogation of the role of lymphatics in kidney biology, there remains much to be uncovered as to how the lymphatic vasculature changes in chronic kidney disease and how they can be manipulated to improve kidney function.

### 2.4 Obesity and metabolic syndrome

Many studies have identified an association between lymphatic dysfunction and metabolic disorders such as obesity and chronic liver disease. This relationship was first documented by the observation that mice lacking Prox1 display adult-onset obesity, increased adiposity, and elevated lipid levels and that restoration of Prox1 in lymphatic endothelium rescued these phenotypes (49, 50). In addition, humans with morbid obesity [body mass index (BMI) > 40] often display impaired lymphatic function and lymphedema, which may become irreversible, even after massive weight loss, in patients with BMI >50 (51, 52).

There is evidence that improving lymphatic function could also ameliorate obesity and its comorbidities by repairing lipid transport and attenuating low-grade inflammation. Disruption of intestinal lacteals, the lymphatic vessels that are critical for dietary lipid absorption and transport into blood stream as chylomicrons, increased the susceptibility to obesity and insulin resistance in mice (53). Conversely, promoting lacteal junction zippering to reduce chylomicron uptake ameliorated diet induced obesity in mice (54, 55). These studies provide evidence that modulation of lymphatic vascular function could be beneficial to control obesity or its comorbidities. Additional molecular mechanisms underlying metabolic disorders and lymphatic dysfunction have been thoroughly explored elsewhere (56–58).

## 3 Preclinical assays to monitor lymphatic function

Several assays have been created to characterize lymphatic vessel function in rodents (Table 1). However, many of these techniques have been used to interrogate mechanisms in lymphedema or lymphatic malformations, which is understandable since the lymphatic vasculature directly contributes to the pathology of these indications (59, 60). Only recently have these assays been applied to mouse models of CKM syndrome. This section describes methods to evaluate lymphatic function in rodents both terminally and non-terminally.

**Table 1:** Preclinical methods to assess lymphatic function in mice.

Target	Description	Endpoints	Advantages	Disadvantages
Peripheral lymphatic imaging—*ex vivo*	Single collecting lymphatic vessels are isolated and imaged directly in the animal or after cannulation	1. Lymphatic contraction frequency, diameter, pressure and output 2. Immune rolling and adhesion	• Direct measurement of lymphatic function • High reproducibility	• Terminal procedure • Low throughput, highly technical
Peripheral lymphatic imaging—*in vivo*	Fluorescent or contrast dye is injected intradermally, allowing visualization of lymphatics and lymph nodes	1. Lymphatic contraction frequency, diameter, pressure and output 2. Total lymphatic clearance 3. Gross lymphatic morphology 4. Vessel permeability 5. Immune rolling and adhesion	• Nonterminal assay, allowing longitudinal analysis • Similar imaging agents are routinely used in the clinic	• Lymphatic contraction may not be representative of overall lymphatic function • Resolution low depending on method • Lymphatic contraction sensitive to heat and level of anesthesia
Immune trafficking	DCs are labeled with fluorescent dyes or transferred from a donor animal, implanted, and quantified in the proximal lymph node or spleen via flow cytometry	1. DC migration from peripheral tissue to lymph node or spleen	• Quantitative measurement of lymphatic immune biology	• Terminal procedure • Directionality in context of disease may be difficult to interpret • Indirect measure of lymphatic function, dependent on several mechanisms
Surgical lymphedema	Major collecting lymphatic vessels are severed in the tail or hindlimb, resulting in lymph accumulation and edema distal to the surgery	1. Tail or limb volume 2. Fibrosis + immune infiltration	• Translationally relevant to pathologies in lymphedema • Endpoints simple to acquire	• Mechanisms may not be relevant to diseases other than lymphedema • Endpoints highly dependent on immediate lymphangiogenic response • Surgeon variability • Longer model
Molecular endpoints	Lymphatic markers (i.e., Prox1, Lyve1, Pdpn) or markers of specific target engagement are measured via qPCR, immunoblot/ELISA, or immunofluorescence	1. mRNA 2. Protein	• Allows quick confirmation of target engagement following therapeutic treatment • Quantitative, high throughput	• Terminal procedure • Reproducibility and signal-to-noise in adult mice unknown • Lymphatics account for minority of tissue mass in most organs

### 3.1 Imaging assays of peripheral lymphatic function

One of the main functions of the lymphatic vascular network is the transport of molecules and fluid from the interstitial space, through proximal lymph nodes, and back toward the venous circulation (1). Muscular tissue contraction and fluid pressure are the main forces responsible for this at the initial lymphatic level (61). However, collecting lymphatic vessels intrinsically produce lymph flow through the contractions of smooth muscle cells (SMCs) lining the vasculature, a process that is dependent on the health of both the SMCs as well as the endothelium (62). Autonomous lymphatic contraction and fluid transport have been used as a readout of overall lymphatic function in mouse models, and several assays have been developed to measure this process. *Ex vivo* methods to study lymphatic contractility include the single-vessel preparation. This procedure entails the isolation and dissection of a lymphatic vessel followed by cannulation in an *ex vivo* perfusion system (63). Vessel pressure and flow can be altered, and compounds can be applied to the bath to examine their functional effects (63). Measurements include vessel internal diameter (end diastolic and end systolic), amplitude, ejection fraction, contraction frequency, and fractional pump flow (63–65). *Ex vivo* preparations allow control of the environment and direct engagement of the lymphatics, which can be helpful in confirming immediate target engagement when therapeutics are directly applied to the vessel.

Although highly quantitative, *ex vivo* preparations of lymphatic vessels lack the surrounding biological components that may govern contraction *in vivo*. Several advances in imaging techniques and injectable tracers have allowed for the visualization and measurement of similar lymphatic parameters in live animals (66). Higher resolution techniques to measure lymphatic function that have potential for clinical translatability include magnetic functional imaging (MRI), photoacoustic (PA) imaging, optical coherence tomography (OCT), and positron emission tomography/computed tomography (PET/CT) (67). Many of these methods are lower throughput, utilize expensive or inflexible tracers, and have limited fields of view in the context of preclinical mouse experimentation (67–72). In recent years, near-infrared fluorescence (NIRF) imaging has gained popularity with the optimization of fluorescent dyes and ease of use. Fluorescein isothiocyanate (FITC)-dextran and indocyanine green (ICG) are two commonly used dyes in this method, with the latter routinely used clinically to visualize lymphatics in patients after surgery or to diagnose primary lymphedema (73). The dye is usually administered intradermally, which fills superficial lymphatics vessels and proximal lymph nodes. The resolution is sufficient to visualize lymphatics that lay closer to skin in mice, such as the collecting lymphatics distal to the popliteal lymph node or within the tail in mice (67). Vessel snapshots or live imaging can be acquired by fluorescence microscopy and used to quantify the rate of lymphatic transport and evaluate lymphatic function (74). High frequency of pigmentation in the C57Bl/6 strain and subcutaneous fat deposition in dysmetabolic mouse models are obstacles to vessel visualization and pose some limitations if this technique is to be used in mouse models of CKM syndrome.

Several nonterminal endpoints can be monitored once the lymphatic vasculature or lymph nodes are visualized utilizing the techniques listed above. Live imaging of the collecting lymphatic vessel allows the measurement of lymphatic function parameters similar to *ex vivo* techniques, such as vessel packet transport, amplitude, and contraction frequency (75). Although these measurements are popularly reported in mouse models and representative of overall lymphatic function, lymphatic contraction frequency is not a discreet clinical parameter and is rarely measured in patients (76, 77). Clinically, fluorescent lymphography is more commonly used as a qualitative measurement, where gross observations are made about morphology and permeability (73). This, too, can be measured in mice, but may lack reproducibility and explicit quantitation (66). Therefore, there is a need for the establishment of a standardized measurement of lymphatic function in patients, which will better guide mouse experimentation. Some examples of imaging endpoints exist that may translate between mouse and human. For instance, pulse-chase style experiments in which a dye or tracer bolus is injected intradermally and imaged live or at time intervals can measure pumping velocity and overall lymphatic clearance (66, 70). Secondly, application of a cuff around the limb (or tail) can allow researchers to calculate lymphatic pressure when combined with live lymphangiography (78, 79). However, a more uniform measurement of lymphatic function in humans would bring more confidence into applying the same measurement for investigating functional mechanisms in mice, which would enable therapeutic discovery.

### 3.2 Immune trafficking assays

A key function of the lymphatic vasculature is to serve as a conduit between immune cells in the periphery and the lymph node. Immune tolerance is tightly regulated by the cross-talk of surveilling DCs in the periphery that acquire and present antigen to T cells, which influence T cell differentiation and activation (80). To do this, the capillary lymphatic endothelium secretes chemokines, such as CCL19 and CCL21, which attract CCR7-expressing DCs and transports them through collecting lymphatic vessels to lymph nodes (8). Lymphatic dysfunction results in overall decreased lymphatic fluid transport, poor DC migration to secondary lymphoid organs, and exacerbated immune dysregulation (81, 82). Several mouse assays have been developed to monitor this crucial process of lymphatics.

A classic method to measure DC migration is through the tracing and quantification of labeled DCs to the lymph node, which can be achieved through several means. DCs can be purified from a donor animal such as Cd45.1+ mice, or mice expressing a fluorescent protein, such as GFP, and transferred to the skin of a recipient model (82, 83). Following transfer, the spleen or lymph node proximal to the area of transfer can be digested, and migrated DCs quantified as Cd45.1^+^ (or GFP^+^)Cd45^+^ Cd11c^hi^MHCII^hi^ by flow cytometry (82, 83). To examine the migration of endogenous DCs, fluorescent macromolecules, such as FITC-conjugated dextran, ovalbumin, or albumin can be directly injected into the lung, tail, or skin, which will be taken up by DCs, and FITC^+^ Cd45^+^ Cd11c^hi^MHCII^hi^ cells can be similarly identified by flow cytometry (84). These pulse-chase style experiments can potentially be performed at the end of a CKM syndrome study and serve as a terminal marker of lymphatic function in mice.

### 3.3 Mouse models of lymphedema

Lymphedema results from direct injury to the lymphatic vasculature and is characterized by the inability of the lymphatic network to clear lymph from distal portions of the body and swelling of the affected area (85). This disease can manifest from either genetic insufficiency (primary lymphedema) or after external insult, such as surgery, chemotherapy, or irradiation therapy (secondary lymphedema) (59, 86). Although lymphedema is a standalone indication, preclinical models of lymphedema allow researchers to interrogate the mechanism-of-action and horsepower of the ability of their target of interest to enhance lymphangiogenesis and overall lymphatic function and could be used to develop and refine preclinical assays to monitor lymphatic function. The Chy mouse model, which contains a loss-of-function mutation in the gene encoding Vegfr3, has been useful in studies of lymphatic dysfunction due its strong lymphatic dysfunction phenotype, including sparse lymphatic coverage, disrupted lymphatic flow, leaky lymphatic vessels, and swelling of the extremities (87). However, this model very closely models primary lymphedema patients and may not be suitable for therapeutic testing on a CKM mouse background. Furthermore, many targets converge on the VEGFR3 signaling axis and would require fully intact VEGFR3 for validation.

The mouse tail lymphedema model has become a commonly used *in vivo* model of secondary lymphedema (88). This surgical procedure requires the circumferential excision of tail skin –2 cm below the base of the tail and injection of Evans Blue dye to visualize the two main collecting lymphatic vessels that run parallel to the lateral veins (89). The lymphatic vessels are then ligated and excised, leading to accumulation of lymph distal to the surgery and swelling until the lymphatic network is reconnected. Edema in this model peaks around three weeks post-surgery, which slowly regresses as lymphatic networks are re-established and fibrous tissue is healed (90, 91). Both collecting lymphatic vessels that run along either side of the tail are commonly excised, but an alternate model has been developed, which excises only one vessel (dominant vessel), enabling functional analysis of the intact vessel (79). The benefit of this model is that the extent of edema is not only dependent on lymphangiogenesis, but also lymphatic output of the remaining vessel, allowing researchers to interrogate a broader spectrum of molecular mechanisms (79).

Aside from tail lymphedema, preclinical models of secondary lymphedema also include the removal of lymphatic vessels in the mouse hindlimb and popliteal lymph node (PLN) dissection (92, 93). However, removal of the PLN alone often does not result in chronic edema (88). Variations of this surgical model have been developed including the removal of several lymph nodes (superficial inguinal lymph node, popliteal lymph node, and deep inguinal lymph node) and excision of the hindlimb femoral lymphatic vessel (94). Both tail and hindlimb methods of inducing lymphedema recapitulate several of the clinical manifestations of prolonged edema, such as dermal thickening, lymphatic vessel expansion and dilation, immune infiltration, and fibrosis (95, 96). Thus, these models could be used to refine the currently available assays for evaluating lymphatic function, establish standardized measurements of lymphatic function between rodent models and patients with lymphedema, and assess the efficacy of therapeutic molecules that directly improve lymphatic function.

### 3.4 Molecular endpoints

Molecular biomarkers such as protein or mRNA that change in response to lymphatic improvement are highly valuable for the monitoring of lymphatic function in preclinical models and eventual clinical trials. Many studies have shown that upon stimulation of lymphangiogenesis or enhanced lymphatic contraction, markers such as *Prox1*, *Pdpn* (podoplanin), *Vegfc* and *Flt4* (gene that encodes VEGFR3) become upregulated, likely due to overall enhanced lymphatic coverage in the tissue of interest, but also through increased transcriptional activity per cell from positive feedback loops (97–99). In addition, increased lymphatic vessel density and lymphatic lumen diameter, which could be visualized through immunofluorescent or whole mount stain, are often observed in rodent models upon stimulation of lymphangiogenesis (26). Furthermore, chemokines such as CCL19 and CCL21 are secreted from functional capillary lymphatics to attract antigen-presenting leukocytes in the parenchyma and could theoretically be used as biomarkers of lymphatic function (100, 101). However, it remains unclear when and to what degree these biomarkers change in adult rodent models of CKM syndrome and which tissue bed would produce the highest signal-to-noise. Many of the examples listed above are analyzed either in development, with chronic treatment of a lymphangiogenic factor or after long-term genetic overexpression or knockout, and how a singular therapy would change the expression of these biomarkers remain unknown (102–104). A similar conundrum occurs in the evaluation of lymphatics in mice through slide mounting and immunofluorescence/immunohistochemistry, where many studies focus on using developmental lymphangiogenesis as a surrogate for improved lymphatic function, while a parameter that can be quantified and clearly correlate with lymphatic function remain to be established (97, 105). Ideally, a clear and specific biomarker should be established so that upon lymphatic enhancement by a drug modality, a gene or protein could be measured within hours or days to confirm activity of the therapeutic and make quick, informed decisions on progressing forward with the study.

In summary, the lymphatic biology field has generated several rodent assays to monitor lymphatic function both terminally and non-terminally. Although some of these lymphatic assays have been used in mouse models of heart failure, atherosclerosis, chronic kidney disease, and obesity, there remains a large gap in knowledge of the level of lymphatic dysfunction in many of the individual disease models. Furthermore, many preclinical models of chronic disease do not develop consistent signs of lymphatic dysfunction that occur in humans, such as pulmonary edema or fluid retention (106, 107). Analyzing a large suite of lymphatic assays in CKM syndrome models may allow head-to-head comparisons of lymphatic contribution to disease.

## 4 Bridging preclinical studies to clinical outcomes in lymphatic anomalies

Many of the preclinical techniques that probe lymphatic function have shown translatability into the clinic and therapeutic advancement. Most of the successes have been made in the field of lymphatic anomalies, which is not unsurprising as this collection of diseases are primarily the direct result of lymphatic dysfunction (60, 108). Lymphatic anomaly is an umbrella term that includes diseases of lymphatic inactivation, such as primary lymphedema, and lymphatic overactivation, such as malformations (59). As mentioned previously, primary lymphedema is caused by a deficiency in the lymphatics, commonly congenital, which can manifest as decreased total body coverage, increased lymphatic leakiness, diminished ability to transport lymph, or enhanced inflammation and fibrosis (98). Primary lymphedema is the result of insufficient lymphatics and lymph absorption, which makes it an enticing indication to translate therapeutic targets into models of CKM syndrome. *FLT4* is a one of the most well-known genetic drivers of primary lymphedema and will be discussed in-depth below in the context of CKM syndrome, but other targets that are causative for primary lymphedema include *VEGFC*, forkhead box protein C2 (*FOXC2*), angiopoietin-2 (*ANGPT2*), cadherin EGF LAG seven-pass G-type receptor 1 (*CELSR1*), and many others that lead to lymphatic valve dysfunction or defective lymphatic endothelial proliferation and survival (109–113). Lymphoscintigraphy utilizing either radiolabeled or fluorescent dyes is the proven method to diagnose primary lymphedema and can be used to quantify lymph velocity and qualitatively monitor the morphology of the lymphatic vasculature for abnormalities or lymph backflow (114, 115). This diagnostic assay has proven preclinical translatability, as discussed above. Unfortunately, primary lymphedema has seen little clinical advancement, with the current standard of care consisting of management of symptoms, manual compression, and surgery (59). This may soon be solved by recent advancements in genetic and diagnostic tools, which have greatly supported successes in lymphatic malformations.

Lymphatic malformations are a collection of lymphatic pathologies characterized by hyperactivation of the lymphatic vasculature. The subcategories include cystic lymphatic malformations (CLM), which manifest as focal lesions, and complex lymphatic anomalies (CLA), which affect multiple organs, including bone, have a wider spectrum of clinical features, and include dysfunction of the major conducting abdominal or thoracic lymphatic vessels (60). Lymphatic malformations are sporadic, making diagnosis and management difficult (60). However, several somatic drivers of the lymphatic malformations have been identified as causative genes, which has greatly advanced understanding of the disease and has led to clinical optimism (108). For example, next generation sequencing has identified somatic gain-of-function mutations in *PIK3CA*, which encodes phosphatidylinositol-4,5,-bisphosphate 3-kinase (PI3K), as causal for cystic lymphatic malformations and a variety of phenotypically distinct complex lymphatic anomalies (116, 117). Mice containing these *Pi3kca* mutations exhibit many of the same pathologies as humans with lymphatic malformations, which were attenuated with alpelisib, a Food and Drug Administration (FDA)-approved PI3K inhibitor for breast cancer patients (102). Alpelisib has shown been shown to reduce lymphatic malformation volume and alleviate symptoms in a small cohort of patients; it is currently ongoing phase II/III testing (NCT05948943) (102). T2-weighted magnetic resonance imaging (MRI) has proven to be the primary method to quantify lymphatic malformation volume in patients and was used to exhibit the efficacy of alpelisib in the *Pik3ca* mouse lymphatic malformation model prior injection into humans, serving as a critical translational tool to be used for target validation (102).

Other ongoing clinical efforts follow a similar paradigm and focus on using FDA-approved a cancer therapies, such as sirolimus and damentib/trametinib drugs to inhibit mTOR and MEK, respectively (108). As mentioned in previous sections, evidence suggests that lymphatic contribution to CKM syndrome may be due to lymphatic vascular insufficiency and rarefaction (23, 24). Interestingly, this is in direct contrast to molecular drivers of lymphatic malformations and suggests that lymphatic health most likely operates on a bell-curve spectrum, where under- or over-activation results in pathology. Therefore, fine-tuning is an important consideration for therapeutic exploration. However, ongoing clinical trials show a clear proof-of-concept that genetic testing and translatable imaging techniques can identify lymphatic drivers of disease and test the efficacy of targeted therapeutics (60, 108). Application of this strategy may allow quick progression of lymphatic-specific therapies in CKM syndrome.

## 5 Therapeutic targets and opportunities

There exist many reports in literature of promising targets that can be modulated to enhance lymphatic function. Several of these studies utilize genetic models to interrogate mechanisms and purely focus on diseases of direct lymphatic dysfunction, such as lymphedema. However, several targets have breached this paradigm and have also been examined in the context of chronic disease. These targets are also amenable to therapeutic development (receptors and enzymes) and have even seen advancement in human clinical trials. We describe the therapeutic opportunity of such targets in CKM syndrome.

### 5.1 VEGFR3

VEGFR3 is a key regulator of lymphangiogensis and function, and thus may be a promising target to enhance lymphatic function in CKM. VEGFR3 is a receptor tyrosine kinase that is enriched in lymphatic endothelial cells in adult animals, which provides specificity to the lymphatic vasculature, thereby sparing the blood endothelium (118). Extracellular binding of its natural ligands VEGFC or VEGFD bring together VEGFR3 homodimers, causing transphosphorylation of the kinase domains and propagating downstream signaling (119). Activation of the protein kinase B (AKT) and Mitogen-activated protein kinase (MAPK) signaling cascades are key nodes by which VEGFR3 activation promotes cellular responses, including lymphangiogenesis and proliferation (120). VEGFR3 may also form heterodimers with vascular endothelial growth factor receptor 2 (VEGFR2) to mediate signaling and angiogenesis, although the relative contribution of this complex in adulthood is not fully understood (121). VEGFR3 is critical to the early development of the lymphatic endothelium, and it also regulates lymphatic function in adulthood (122). Clinically, VEGFR3 loss-of-function mutations leads to primary lymphedema, most notably Milroy disease (123).

While VEGFC can engage both VEGFR2 and VEGFR3, an engineered point mutation in VEGFC (cysteine 156 to serine, VEGFC_C156S_), was found to abolish binding of VEGFC to VEGFR2 and selectively activate VEGFR3 (31). VEGFC_C156S_ maintains lymphatic activation of VEGFR3, stimulates lymphangiogenesis *in vivo* and eliminates possible effects of VEGFC binding to the blood endothelium (26). There is clear evidence that VEGFC_C156S_ improves cardiac function in the context of acute cardiac injury, such as myocardial infarction (MI). Numerous preclinical studies showed that VEGFC_C156S_ treatment increased lymphangiogenesis, reduced cardiac congestion, decreased inflammation, attenuated fibrosis, and rescued cardiac dysfunction in mouse or rat coronary artery ligation and/or occlusion models of MI (25, 124–126). Notably, VEGFR3 inhibition via the kinase inhibitor MAZ51, soluble VEGFR3, or VEGFR3 blocking antibodies exacerbated cardiac dysfunction and disease pathogenesis (27). However, lymphangiogenesis blockade in heart using *Flt4* knockout or *Vegfc/Vegfd* double knockout animals did not exacerbate cardiac dysfunction after myocardial infarction (MI), indicating that the beneficial effects observed with VEGFC_C156S_ in MI could be mediated by alternative mechanisms (127).

In addition to acute cardiac injury, VEGFC_C156S_ treatment is also effective in chronic heart failure preclinical models. In a six-week angiotensin II-induced model of hypertension and systolic dysfunction, VEGFC_C156S_ improved ejection fraction, lowered blood pressure, and reduced inflammation (128). Consistent with this result, in a six-week chronic heart failure model generated by pressure overload, VEGFC_C156S_ alleviated cardiac dysfunction, fibrosis, inflammation and edema (28).

Furthermore, VEGFR3 activation through VEGFC_C156S_ administration alleviated fibrosis and inflammation in a CKD model induced by unilateral ureteral obstruction, and improved cystic disease and reduced inflammation in mouse models of ADPKD (40, 129). Moreover, VEGFR3 is expressed in human glomerular endothelial cells and attenuates VEGFR2 phosphorylation upon VEGFA stimulation, implicating a potential role for VEGFR3 in regulating glomerular filtration barrier function (130). In contrast, Vegfr3 inhibition using soluble VEGFR3, anti-VEGFR3 antibodies or transgenic Vegfr3 overexpression approach demonstrated beneficial effects in multiple clinical models of AKI (131). The context-dependent role of VEGFR3 in kidney diseases warrants further investigation.

Despite the extensive preclinical evidence that VEGFR3 activation may be beneficial in diseases with lymphatic dysfunction, only a few attempts have been made at translating this pathway into the clinic. One consideration for targeting VEGFR3 clinically may be safety concerns associated with chronic activation of a growth factor signaling pathway, such as enhanced vessel leakiness, or potentially carcinogenesis risk. This topic will be discussed further below. Secondly, questions remain regarding the horsepower of lymphatic vascular targeting to significantly improve CKM. Lastly, while VEGFR3 is enriched in the lymphatic endothelium, it can also be expressed in the bone as well as liver sinusoidal endothelial cells (104). VEGFR3 activation in these cell types may produce unintended consequences.

One method to overcome these potential risks includes targeted VEGFC gene delivery. Lymfactin is adenoviral-mediated VEGFC gene delivery, which is administered to breast cancer patients with high risk for upper extremity lymphedema via a lymph node flap that is collected from the groin area and treated *ex vivo* (132). The lymph node flap was then applied to area likely to undergo lymphedema. This therapy stimulated lymphatic growth and enhanced lymphatic flow in porcine preclinical studies (133). In humans, lymph node transfer and Lymfactin treatment was well tolerated and exhibited a 46% reduction in excess arm volume after a 24 month follow up (134). Targeted therapies such as this would be more challenging in CKM disorders, which are likely systemic diseases. Technological advancements will be necessary to increase the number of patients who would benefit from lymphatic-targeted therapies.

### 5.2 Adrenomedullin

Another growth factor that is important for lymphatic function and growth is adrenomedullin (AM). AM is a hormone peptide that enacts its signaling via binding to the G protein-coupled receptor calcitonin receptor-like receptor (CLR) in complex with either receptor activity modifying protein (RAMP) 2 or 3 (135). AM predominantly activates the Gs alpha signaling cascade, which directly increases intracellular cyclic AMP to transduce downstream signaling (136). CLR-RAMP2/3 complexes are found in many tissue types, and the biological effects of AM signaling depends on the cell type activated. Of note, AM can activate angiogenesis pathways in both blood and lymphatic endothelial cells (137). AM regulates vascular tone via downstream endothelial nitric oxide synthase (eNOS) activation, which increases vasodilation and endothelial barrier function in the cardiovascular system (138, 139).

AM is clearly associated with lymphatic health, as deletion of the gene encoding adrenomedullin (*Adm*), calcitonin receptor-like receptor (*Calcrl*), and *Ramp2* all resulted in interstitial lymphedema, abnormal lymphatic vessels, and embryonic lethality (140). This finding was confirmed in adult mice, where *Adm* haploinsufficiency caused lymphedema in the hindlimbs after skin incision (141). Pharmacological delivery of AM via osmotic minipump attenuated tail swelling in a mouse model of surgically induced tail lymphedema (142). Furthermore, genetic AM overexpression improved cardiac function and decreased edema in a left anterior descending coronary artery ligation mouse model of myocardial infarction (143). Overall, AM signaling may be a potent mechanism to enhance lymphatic function and health in CKM.

Researchers have explored efficacy of enhancing AM signaling in the clinic due to its reported anti-inflammatory and anti-microbial effects (144). Adrecizumab is a humanized monoclonal antibody that binds and stabilizes AM without interfering with CLR engagement, which extends the normally short terminal half-life of AM to fourteen days (145). Adrecizumab has been used in clinical studies to test efficacy in sepsis, where it was reportedly safe and well-tolerated. In this study, adrecizumab improved organ function and significantly reduced patient mortality (146). Adrecizumab is also currently being tested clinically for acute heart failure as a proof of principle study that may lead to future studies in chronic heart failure (NCT04252937). Additional methods for overcoming the short half-life of AM involve continuous AM infusion or AM modification by PEGylation (147). Lastly, AM is displayed a protective role in kidney disease through its vasodilatory, natriuretic, and diuretic actions (148, 149). Combined with AM's reported ability to attenuate adipose inflammation and work synergistically with glucagon-like peptide 1 (GLP-1) in appetite regulation, AM has promise as a therapeutic target for CKM (148–151). Although the AM/CLR/RAMP signaling axis is complex, the specificity for receptor combinations that lead to certain biologic responses may allow researchers to develop more targeted therapeutics to interrogate this pathway.

### 5.3 Eicosanoids

Eicosanoids are lipid signaling molecules originally derived from arachidonic acid and polyunsaturated fatty acids that have a diverse set of functions, most notably in modulation of immune function and inflammatory processes (152). Leukotriene B4 (LTB_4_) is a bioactive lipid generated from the 5-lipoxygenase (5-LOX) branch of eicosanoid metabolism that exerts its biological effects via binding to its cognate LTB_4_ receptors, (LTB4Rs), and promoting g-protein signaling (153, 154). LTB_4_ core function includes leukocyte activation and pro-inflammatory signaling in the endothelium (155). LTB_4_ antagonism via ketoprofen significantly reduced tail swelling and pathogenesis in a mouse surgical tail lymphedema model (156). In pathogenic conditions, such as lymphedema, the authors found that LTB_4_ concentrations rose to a level that inhibits lymphatic coverage and worsens disease outcomes. Interestingly, this mechanism was dependent on Vegfr3 and Notch1, indicating that many lymphatic endothelial pathways converge on similar molecular targets (156).

LTB4 inhibition revealed promising preclinical efficacy in rodent models of lymphedema, which spurred a two-part clinical study to test effectiveness and safety in patients. In the open-label portion of the clinical study, patients with primary or secondary lymphedema were given oral ketoprofen three times a day for four months (157). After the four-month period, patient histopathology score and skin thickness were significantly reduced compared to baseline. In the second part of the study, a double-blind, placebo-controlled experiment, patients receiving ketoprofen had significantly reduced skin thickness, histopathology score, and circulating inflammatory marker expression compared to baseline than patients receiving placebo (157). Notably, ketoprofen also inhibits cyclooxygenases (COX), and pro-inflammatory signaling by these enzymes may contribute to the overall horsepower of these clinical results (158).

Prostaglandins generated by the COX arm of eicosanoid metabolism are also important for lymphatic function and regulation of CKM. One study found that COX2-generated lipid mediators and Vegfr3 signaling were responsible for high-fat diet-induced metabolic dysfunction in mice and erratic mesenteric lymphatic vessel growth (159). This led to unregulated leakage of fat-concentrated lymph into adipose, which worsened metabolic dysfunction. The authors of this study chemically modified the COX2 inhibitor celecoxib to increase targeting specifically to the mesenteric lymph and increase its bioavailability. This modified prodrug successfully blocked prostaglandins within the mesenteric lymph, decreased Vegfc levels, inhibited lymphatic leakage, and attenuated metabolic dysfunction (159). This study reveals the complex feedback mechanisms surrounding Vegfr3 in disease but serves as an important proof-of-concept that amelioration of lymphatic dysfunction may provide significant benefits in chronic diseases such as obesity or CKM.

### 5.4 Other pathways with early evidence for further investigation

In addition to VEGFR3, adrenomedullin, and eicosanoids, several other targets and pathways have also been reported to regulate lymphangiogenesis and lymphatic function. For example, angiopoietin 2 (Ang2) plays important role in regulating lymphatic vessel development and function. Ang2 is an antagonist ligand for TEK receptor tyrosine kinase (TIE2) receptor in blood endothelium, where the vascular endothelial protein tyrosine phosphatase (VE-PTP/PTPRβ) is expressed, but is an agonist ligand for TIE2 in lymphatic endothelium, where VE-PTP expression is lacking (160). Ang2-Tie2 signaling in the lymphatic endothelium is required for Vegfr3 expression and signaling as well as Vegfc-induced lymphangiogenesis in adult mice, and blocking Ang2 using antibodies reduced Vegfr3 expression and inhibited lymphangiogenesis, highlighting the important role of Angiopoietin-Tie2 signaling in the lymphatic endothelium (161–163). In addition, Epsins also regulate Vegfr3 degradation in the lymphatic endothelium. Lymphatic-specific Epsin knockout alleviated Vegfr3 degradation and subsequent inhibition of lymphangiogenesis in diabetic mice, suggesting that modulation of Vegfr3 localization could be a potential therapeutic approach to restore impaired lymphangiogenesis (91). Furthermore, several other lymphangiogenic factors, such as collagen and calcium-binding EGF domain-containing protein (CCBE1), Semaphorins and Neuropilins, fibroblast growth factor-2 (FGF2), sphingosine 1-phosphate (S1P), bone morphogenetic protein-9 (BMP9) and activin-like kinase receptor type I (ALK1), Notch1 and Ephrin B2 are studied and reviewed thoroughly elsewhere (164). In addition to lymphangiogenic factors, lymphoangiocrine molecules, such as Reelin, that are secreted from the lymphatics during injury are reported to mediate the beneficial effects of lymphangiogenesis in a disease context, and these factors and associated pathways could be worthwhile to investigate as novel therapeutic targets (165, 166).

## 6 Safety considerations

Although there is strong preclinical evidence that the enhancement of lymphatic function will lead to efficacy in disease outcomes, there exist many unknowns about safety and tolerability for several of the above-mentioned therapeutic opportunities. Firstly, most of the targets that enhance lymphatic function also classically activate lymphatic endothelial proliferation and lymphangiogenesis. Overactivation of this pathway may have potential oncogenic off-target effects. Tumors can express several lymphatic mitogenic factors, including VEGFC and AM, which correlate with disease severity (167, 168). These factors can play a role in tumor vascularization, survival, and metastasis (169, 170). Furthermore, there may be opposing biological functions of increasing lymphangiogenesis via classical mechanisms. One example of this is the regulation of vascular barrier function and angiogenesis by the VEGFA signaling pathway, which enhances vascular proliferation and angiogenesis and is simultaneously, a potent activator of vascular permeability (171, 172). VEGFC has pro-lymphangiogenic effects and may worsen the lymphatic barrier at certain concentrations or in specific disease settings (159). However, this pathway also reportedly enhances endothelial barrier function, which indicates that a balance may needed when targeting VEGFC therapeutically (173, 174). Thus, further investigation is needed to identify differences between VEGFA and VEGFC biological activity in the blood endothelium (173). Several targets, such as AM, LTB4, and COX2 activate pathways that are ubiquitously expressed in several other tissues, which could result in unwanted or unexpected outcomes, especially in a chronic dosing scenario. Furthermore, inhibition of classical inflammatory responses may increase risk for infection and could be detrimental in disease resolution. Methods to specifically target the lymphatic endothelium, such as antibody-drug conjugate (ADC), nanoparticles, or local lymph delivery may provide specificity to test mechanism of action while enhancing the therapeutic safety profile.

## 7 Conclusion

Enhancement of lymphatic function has the potential to bridge systemic and organ-specific pathologies within CKM syndrome. Restoration of lymphatic function can stabilize metabolite trafficking and attenuate inflammatory processes in metabolic disorders (98, 175). Furthermore, lymphatic restoration has the potential to simultaneously improve cardiac and kidney parameters directly. However, the role of lymphatics in human disease has only recently come into appreciation, and much remains unknown in different indications and patient populations. Animal models displaying key aspects of human CKM syndrome, such as edema, are also lacking, which makes identifying novel therapeutic mechanisms targeting lymphatic function challenging. Rodent models of atherosclerosis, heart failure, and kidney disease are continuing to be refined and optimized, and a complete picture of lymphatic function, from lymphatic contraction to DC trafficking, would help bring a better understanding of the role of lymphatics in these indications.

Although technological advances allow researchers to analyze lymphatic function with high resolution and fidelity, there are not yet any FDA-approved therapies to improve lymphatic function, even in lymphatic-specific diseases, such as lymphedema. One major challenge is specificity. The lymphatic vascular network differs greatly in function from the blood vasculature, yet many therapeutic targets may affect both (1). An ideal therapeutic target would be unique to lymphatic cells or lymph fluid and important in physiology or disease. To this end, the composition of lymph fluid, both in human disease and preclinical models of disease, has been widely understudied, in part due to difficulty in sampling in mice. How the lymph differs from the blood during pathogenesis may provide insights into specific pathways up- or down-regulated in the lymphatics. Furthermore, many preclinical mechanisms propose improvement of lymphatic coverage through enhanced lymphangiogenesis (176). Whether functional lymphangiogenesis can occur in adult humans, and if this can enhance fluid or immune clearance, is currently speculative. Nonetheless, significant progress has been made in both the understanding of lymphatics and CKM syndrome, and several targets have the promise for further testing in humans. Further study and refinement may one day identify a specific and safe lymphatic therapy with the potential to greatly benefit patient outcomes.
